# Understanding Lignin Radical Dynamics: Quenching Radicals by Solvent and Thermal Induced Mobility

**DOI:** 10.1021/acs.biomac.6c00744

**Published:** 2026-06-29

**Authors:** Åke Henrik-Klemens, Liam Mistry, Anette Larsson

**Affiliations:** 1 Applied Chemistry, Chemistry and Chemical Engineering, 11248 Chalmers University of Technology, SE-412 96 Gothenburg, Sweden; 2 Wallenberg Wood Science Center, Chalmers University of Technology, SE-412 96 Gothenburg, Sweden; 3 FibRe, Centre for Lignocellulose-Based Thermoplastics, Department of Chemistry and Chemical Engineering, Chalmers University of Technology, SE-412 96 Gothenburg, Sweden

## Abstract

Lignin contains persistent free radicals (PFRs) stabilized by restricted molecular mobility in a glassy state. Herein, we investigate quenching effects in softwood kraft lignin PFRs by solvation and thermal treatment. Electron paramagnetic resonance (EPR) spectroscopy and temperature-modulated differential scanning calorimetry (TM-DSC) were used to characterize changes. Room-temperature methanol or acetone swelling reduced the paramagnetic signal to 48% and 71% of the original intensity. Similarly, heating lignin above its glass transition temperature (*T*_g_) mobilized trapped radicals, leading to their recombination and the depletion of the EPR signal. Both heating and solvent swelling induced a transition from carbon- to oxygen-centered radicals (*g*-value 2.0016 → 2.0033), but only heating caused cross-linking that increased the *T*_g_ by approximately 15 °C. Ultimately, overcoming the restricted mobility of the glassy state, whether chemically or thermally, is the primary driver for quenching persistent radicals in kraft lignin.

## Graphical abstract

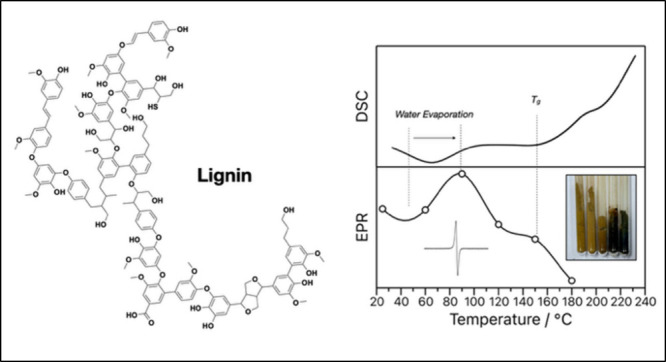

## Introduction

The phenolic, rigid structure of lignin makes it a stable radical carrier. Lignin radicals originate both from their polymerization and from natural oxidative processes during aging and decay; in the case of kraft lignin (KL), additional radicals are introduced during the pulping process. In the glassy state, these radicals are persistent due to restricted molecular mobility, which limits lignin–lignin termination reactions as well as oxygen diffusion.^1^

However, when lignin is heated to temperatures slightly above its glass transition temperature (*T*_g_), its physicochemical behavior changes markedly. In this regime, oxygen gas is consumed,^2^ and the material’s overall oxygen content increases.^3^ In addition, a net exothermic heat flow is typically observed just above *T*_g_,^4–6^ accompanied by an increase in molecular weight.^3,5^ Electron paramagnetic resonance (EPR) measurement by Hatakeyama and Nakano (1970) indeed found that the net radical content of the lignins were reduced when heated above their *T*_g_, even if it was also evident that new radicals formed as well.^7^ Together, these observations suggest that persistent free radicals (PFRs) in lignin matrix become activated by the mobility achieved just above the *T*_g_, promoting oxidative reactions and radical coupling that led to cross-linking. Hatakeyama and Nakano (1970) also found that dissolving γ radiated lignin model compounds reduced their radical signal; however, the effect on swelling lignins has yet to be investigated.^7^

While lignin mobility can be enhanced through swelling or dissolution in solvents, the effect of these specific mobility changes on lignin radical content has not yet been systematically investigated. The literature to date has primarily focused on the chemical scavenging of radicals rather than physical mobility effects. For example, Voitl and Rudolf von Rohr established that methanol can “deplete” radicals by generating methyl (·CH_3_) and methoxyl (·OCH_3_) radicals that cap reactive lignin fragments.^8,9^ It is also known that the physical detection of this depletion via EPR is heavily influenced by the solvent (e.g., methanol vs water) and its ability to stabilize or quench phenoxyl radicals (often termed “·OH scavenging”).^10–12^ Additionally, parallel mechanisms like nucleophilic trapping have been described by Gierer et al., where alcohols undergo nucleophilic attack on quinone methide intermediates to halt condensation.^13^ To move beyond these isolated chemical mechanisms and understand the overarching role of mobility, this work employs electron paramagnetic resonance (EPR) spectroscopy. We systematically examine how increased molecular mobility in softwood kraft lignin, achieved either by heating above its *T*_g_ or via solvent swelling, alters its radical content. Finally, we assess how these radical-mediated processes influence the lignin’s reactivity and *T*_g_ using temperature-modulated differential scanning calorimetry (TM-DSC).

## Experimental Section

### Materials and Sample Preparation

All chemicals and reagents used in this study were of analytical grade and obtained from Sigma-Aldrich. A softwood LignoBoost kraft lignin supplied by a Swedish pulp mill was used in this work. We have previously characterized both its molecular as well as thermal properties previously; including molecular weight, chemical compositions and thermal characteristics.^4^ The lignin was swollen by adding acetone or methanol to solvent lignin ratio of 3:1 in glass vials. After 24 h, the solvent was allowed to evaporate. Once the lignin was dry, it was put in a vacuum (3 mbar) for 1 h to remove any remaining solvent.

### Calorimetry

The temperature modulated DSC technique of TOPEM was employed on a DSC 5 STARe system instrument (Mettler Toledo) in 70 μL aluminum crucibles. To isolate the contribution of residual moisture from cross-linking, a subset of KL samples was preannealed at 80 °C for 5 min prior to measurement. Measurements of MeOH-KL and Acetone-KL were performed on dried powders after solvent evaporation and vacuum treatment, as described in the Sample Preparation section. All samples were run in the first heating cycle unless stated otherwise; second heating cycles were performed on KL only to assess thermal history effects. The samples were subjected to one or two heating ramps from 25 to 230 °C (2 °C/min), with a 20 °C/min cooling ramp in between. The temperature modulation was applied with an amplitude of 0.5 °C/min and a pulse of 30–45 s. The *T*_g_ was determined as the midpoint between two baselines.

### Spectroscopy

Electron paramagnetic resonance (EPR) spectroscopy measurements were conducted using a SpinScan X spectrometer from LINEV Systems, operating in X-band mode (9.4 GHz). Prior to each experiment, the spectrometer was calibrated using a Mn^2+^ standard (see Supporting Information).

For each measurement, approximately 20 mg of the prepared sample was placed in a Wilmad NMR tube (5 mm diameter) and positioned within the resonant cavity of the spectrometer. Samples were measured at a room temperature of (cavity temperature: 305.15 K) throughout the analysis.

Thermal treatment of lignin was performed using the proprietary temperature control module using N_2_ carrier gas. Samples were held at the target temperature (25, 60, 90, 120, 150, and 180 °C) in sequence for 5 min to ensure thermal equilibration, after which they were allowed to cool back to room temperature for measurement. This is important to ensure consistency, avoiding variations in instrument sensitivity, dielectric losses, and line broadening effects associated with in situ heating.

### Data Acquisition

Our X-band EPR spectrometer was configured with the following parameters to optimize the detection of radical species in the lignocellulosic samples:

- Microwave power: 27 dB
- Modulation frequency: 100 kHz
- Modulation amplitude: 100 μT
- Magnetic field sweep width: 337 mT (±15)
- Sweep rate: 8.0 mT/s

Data were acquired and processed using eSpinoza (version 1.1.0.2) software. All collected spectra were baseline-corrected before further analysis or fitting.

## Results and Discussion

EPR spectroscopy has been widely employed to characterize and quantify the persistent free radical (PFR) content in lignin.^1^ While lignin radicals are often broadly described as phenoxy-type (yielding narrow, isotropic-looking signals without resolved hyperfine structure), their specific atomic nature can be further elucidated via their *g*-values. Herein, X-band EPR was used to compare LignoBoost softwood kraft lignin (KL) with methanol-swollen (protic, MeOH-KL) and acetone-swollen (aprotic, Acetone-KL) samples as dried powders (Figure 1a). Under identical room-temperature conditions, the paramagnetic signal for MeOH-KL and Acetone-KL depleted by 48% and 71%, respectively, when compared to untreated KL. There was a distinctive color change following solvent treatment, from the light-brown typical of KL to a darker-brown as highlighted in Figure 1a inset. Drying lignins under different conditions was shown by Zhang et al. to affect color based on microaggregations.^14^ The notably higher quenching efficiency observed in the aprotic solvent (acetone) are consistent with a dominant role for physical mobility and macromolecular swelling, though a contribution from differential solvent–radical interactions cannot be fully excluded without further spectroscopic characterization. While methanol can participate in chemical scavenging via hydrogen atom transfer (HAT), its lower depletion rate compared to acetone implies that the superior solubility and swelling power of acetone in softwood kraft lignin provides a lower barrier for radical–radical recombination.^15^ Consistent with this, kraft lignin is known to adopt more expanded conformations (larger radius of gyration, *R*_g_) in aprotic solvents such as THF and acetone than in methanol, where intermolecular hydrogen bonding promotes compact aggregation.^15,16^ Greater chain extension would directly increase the probability of radical–radical encounter within the swollen matrix. This indicates that in the liquid phase, the physical “loosening” of the glassy lignin matrix is a more potent driver for radical annihilation than active protic scavenging alone.

**Figure 1:**
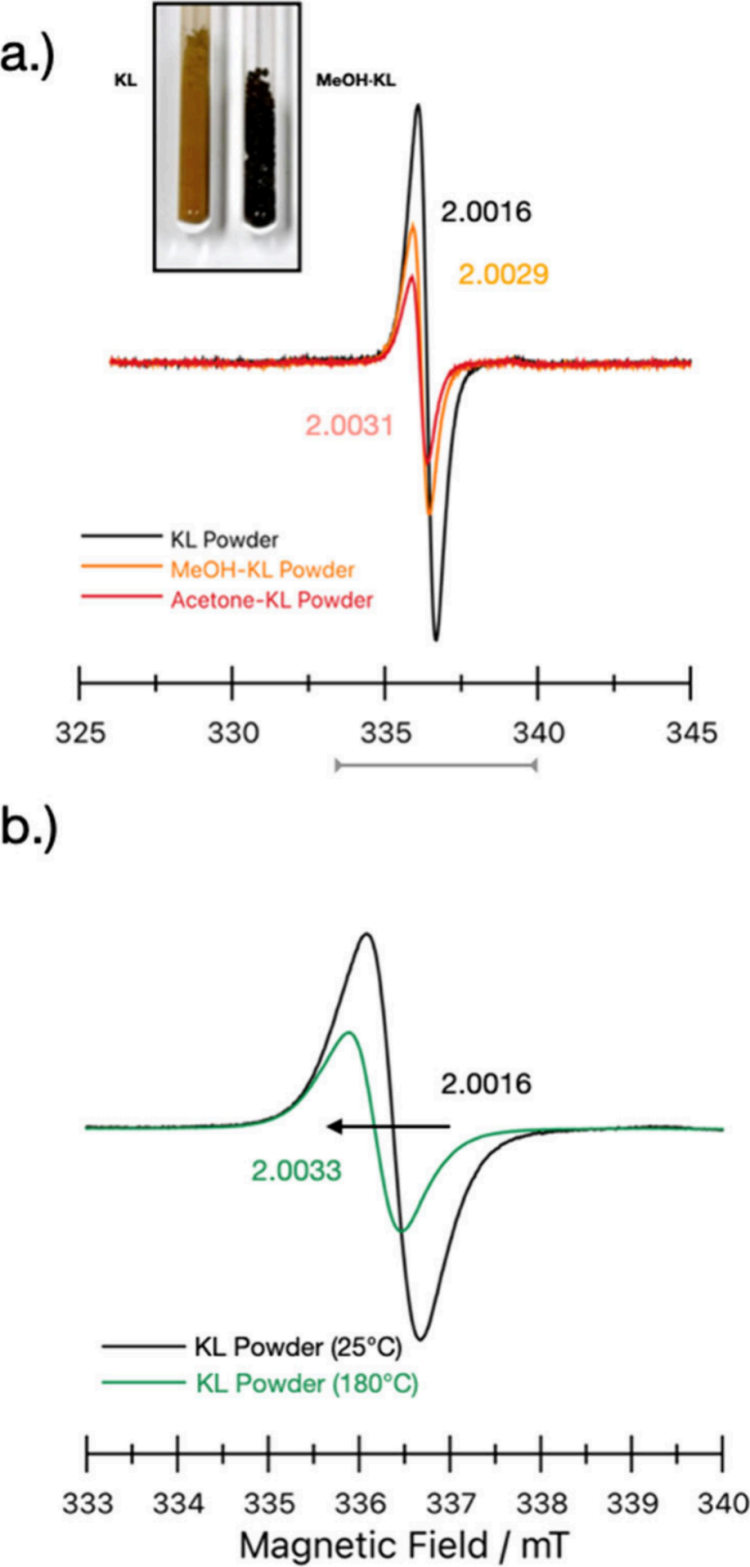
X-band EPR spectra of (a) standard (black line), methanol-treated (orange line), and acetone-treated (red line) kraft lignin as dry powders at room temperature. The inset highlights the visual color difference between the standard and methanol-treated lignin. (b) EPR spectra demonstrating a shift in *g*-value and a decrease in signal intensity when standard kraft lignin is heated from 25 °C (black line) to 180 °C (green line).

The recorded *g*-values (2.0016–2.0031) for all KL samples at room temperature remained similar to that of a free electron (2.0023), typical for lignin (KL, 2.0016; MeOH-KL, 2.0029; Acetone-KL, 2.0031).^1,17^ Values around 2.003 are characteristic of carbon-centered organic radicals delocalized over an aromatic structure, implying that while the solvents effectively quench a large portion of the radicals, the remaining species share similar electron environments.^18^ Thermal treatment of standard KL to 180 °C (Figure 1b) shifted the *g*-value from 2.0016 to 2.0033 alongside a significant drop in signal intensity. This thermal treatment narrows the line width and aligns the *g*-value more closely with those of the solvent-treated KL samples, indicating that increased mobility, whether solvent-induced or thermally driven above the *T*_g_, results in a more uniform population of remaining radicals within the lignin matrix when compared to methanol or acetone treated KL. This aspect will be explored in more detail below.

The *T*_g_ in DSC is typically determined from the second heating run to eliminate the effects of moisture and prior thermal history. Water is problematic for lignin, as it acts as a plasticizer, lowering the *T*_g_. In addition, the endothermic evaporation of water can obscure the relatively small change in heat capacity associated with the glass transition of lignin.^19^

In the present work, however, the objective was to investigate processes occurring in untreated material. Therefore, temperature-modulated DSC (TM-DSC) was employed, which separates contributions to the total heat flow into reversing (Figure 2a) and nonreversing (Figure 2b) components. Processes that affect the heat capacity, such as the glass transition, appear in the reversing heat flow, whereas kinetic events such as water evaporation and chemical reactions appear in the nonreversing heat flow.^20^ This separation allows both sets of processes to be evaluated independently.

**Figure 2:**
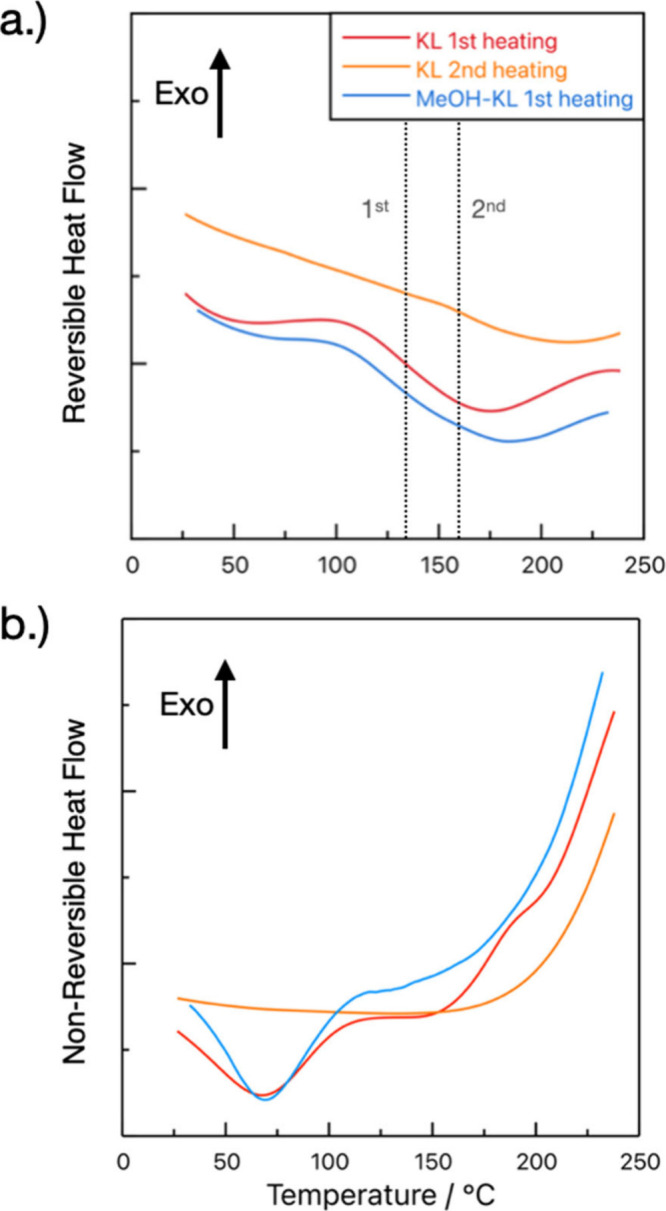
TM-DSC curves of treated (MeOH) and nontreated kraft lignin for (a) reversible and (b) nonreversable heat flow. Vertical dashed lines at 141 °C (first heating *T*_g_) and 165 °C (second heating *T*_g_) added for clarity.

For KL in the first heating cycle, the first event is water evaporation (50–100 °C, nonreversing), followed by the glass transition, which has its midpoint at 141 °C (reversing), and then around 150 °C, exothermic processes start taking place (nonreversing). At 180 °C there is a shoulder in the nonreversing, indicating the end or ebbing of an exothermic process.

For MeOH-KL the curves look similar but without the shoulder at 180 °C in the nonreversing, indicating that this feature is related to the loss in radical content. Likewise, in the second heating of KL, this shoulder is also gone. However, the *T*_g_ does not change significantly upon swelling KL in MeOH, but the *T*_g_ of KL in the second heating was significantly increased by approximately 15 °C. It is possible that this is a consequence of losing the plasticizing water or of cross-linking or both. To further investigate this increase in *T*_g_, KL and MeOH-KL were annealed at 80 °C for 5 min to remove residual moisture. The resulting *T*_g_ in the first run after cooling to room temperature was intermediate, indicating that the observed discrepancy cannot be attributed solely to the plasticizing effect of water but also involves cross-linking reactions. Thus, the first heating cycle induces more pronounced chemical changes than swelling KL in methanol, even though the reduction in radical content was of similar magnitudes. The *T*_g_ of KL and MeOH-KL is not significantly different after annealing, further indicating that the radical reduction upon swelling the lignin does not lead to substantial chemical changes. These results are presented in Table 1 below.

**Table 1:** *T*_g_ Determined from the Reversing Heat Flow

sample	heating cycle	***T***_g_ (*n* = 3)
MeOH·KL	1st	141 (±2)
KL	1st	141 (±2)
KL	1st (with annealing)	154 (±2)
MeOH·KL	1st (with annealing)	156 (±1)
KL	2nd	165 (±4)

The aforementioned DSC results permitted us to explore EPR as a function of temperature (Figure 3) by monitoring the thermal response of kraft lignin up to and above the *T*_g_.

**Figure 3:**
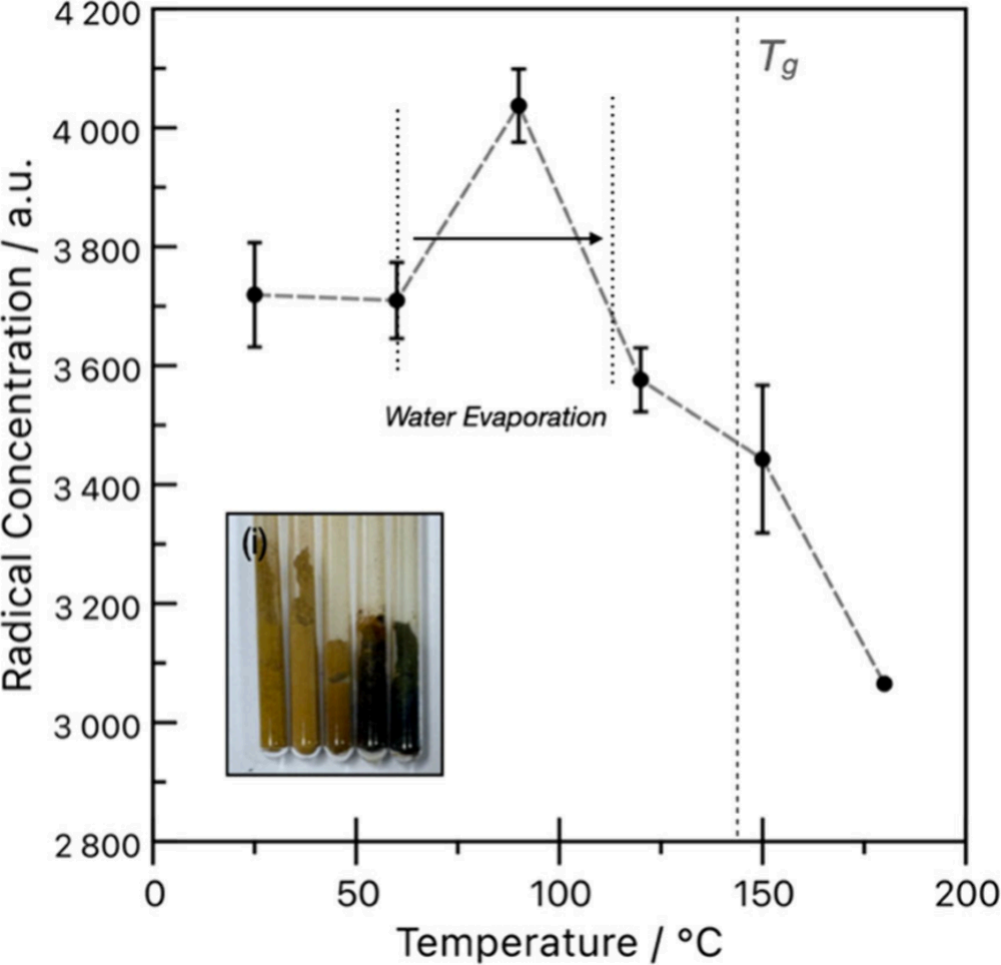
Temperature-dependent EPR of KL lignin measured by X-band EPR spectroscopy. Measurements were made in quintuplicate series (average SD: ±132). Inset (i) photo highlighting the discoloration after thermal treatments (left to right: 60, 90, 120, 150, and 180 °C). The glass transition temperature reference line is at 141 °C.

The DSC trace shows an initial endothermic event between ∼50 and 100 °C, attributed to the evaporation of residual moisture. EPR also presented modest increase in signal intensity up to ∼80 °C and initially linked to the same loss of water, thus a reduced quenching effect. Upon further heating, a broad increase in heat flow was observed, culminating near the *T*_g_, indicated by the inflection around ∼140 °C. The EPR-derived radical concentration exhibits a clear temperature dependence. This is observed in EPR as a gradual decline or depletion of the signal intensity is observed as the material transitions toward and beyond *T*_g_. This is consistent with thermally driven radical quenching due to increased structural mobility allowing radicals to move and collide, leading to recombination or reactions with other functional groups, effectively annihilating the EPR signal. Below 100 °C radicals are trapped within a glassy state within the rigid polymer matrix where this occurs slowly. Together, these data sets highlight a strong correlation between thermal softening of the lignin matrix and the loss of EPR-active species.

Concurrently, the *g*-value shifted from 2.0016 to 2.0033 (Figure 4), as alluded to in Figure 1b, marking a change in lignin character from one dominated by carbon-centered radicals (CCR) to one with greater oxygen-centered, phenoxyl-type radicals (often termed oxygen-centered semiquinone free radicals (SFR)). These changes could possibly be generated through the thermal cleavage of weak aryl ether linkages, such as the β-O-4 or α-O-4 ether, or by reactions with molecular oxygen which diffuses faster in the mobile matrix. As oxygen has a larger spin–orbit coupling constant than carbon, the unpaired electron’s proximity to the oxygen atom shifts the *g*-value higher. In organic EPR spectroscopy, carbon-centered radicals exhibit *g*-values of 2.003, whereas oxygen-centered radicals (like the phenoxyl- or semiquinone radicals often found in lignin) typically exhibit *g*-values of 2.004.^18,21^ These findings reflect molecular mobility and evolving electronic environments around the radical species as the lignin softens at elevated temperatures. It remains unclear if the populations of specific types of radicals are disproportionately affected by temperature, or if CCRs transition to SFRs.^22^ We speculate that a combination of the two occurs at different rates, but it remains clear that a deeper understanding of the radical formation and depletions mechanisms is needed. Open questions remain regarding the absolute radical concentration per unit molecular weight in KL and the intramolecular localization of radical species, whether preferentially at chain ends, branch points, or within the β-O-4-rich backbone. High-field EPR or EPR imaging methods, combined with model compound studies, represent promising routes to resolve these questions in future work.

**Figure 4:**
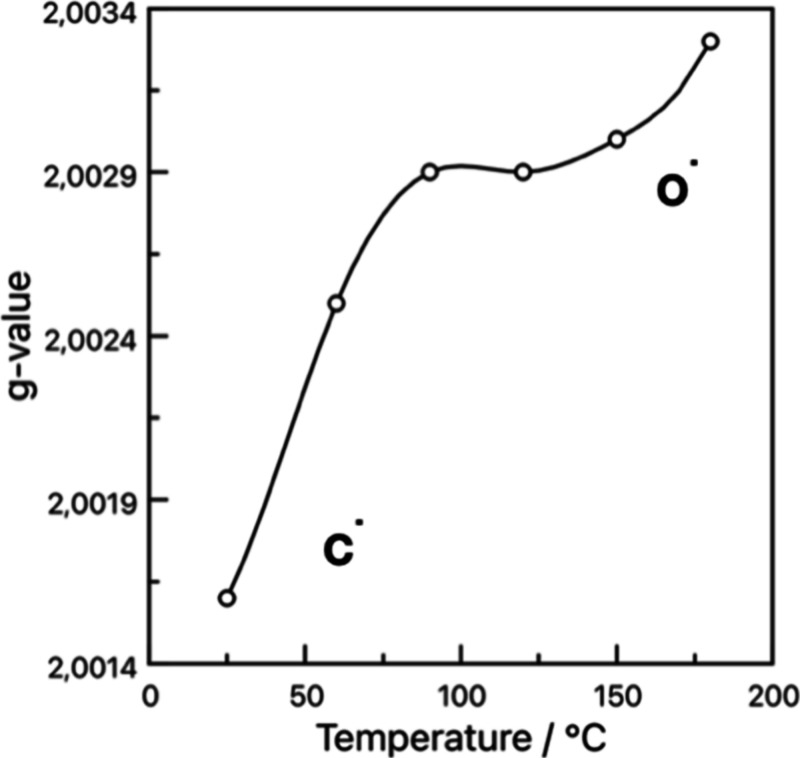
Temperature dependence of the *g*-value for kraft lignin measured using X-band EPR.

The sharp onset of radical depletion above ∼140°C is consistent with the well-established rapid increase in fractional free volume above *T*_g_, which provides the translational mobility necessary for radical–radical recombination and scales with (*T* – *T*_g_) according to free-volume theory.^23^ Below *T*_g_, the fractional free volume is effectively frozen, confining radicals within cage-like domains of the dense glassy matrix and preventing the diffusion-limited encounters required for recombination, a mechanism well-established for persistent radicals in glassy organic polymers.^23,24^ Above *T*_g_, the simultaneous liberation of free volume and onset of segmental chain mobility open two competing pathways: homolytic radical–radical recombination and oxygen-mediated oxidation, the latter facilitated by the increased diffusivity of O_2_ through the softened matrix. The observed *g*-value shift toward oxygen-centered character is consistent with the preferential loss of sterically hindered carbon-centered radicals, which require greater molecular mobility to recombine, leaving a residual population enriched in phenoxyl-type species.^20,23^

## Conclusions

In summary, this study demonstrates that the restricted molecular mobility of the glassy state is the primary factor stabilizing persistent free radicals (PFRs) in kraft lignin, in agreement with the established literature. By systematically increasing macromolecular mobility through either thermal activation or chemical solvation, we observed significant radical quenching, although the underlying mechanisms depend on the mode of activation. The superior quenching efficiency of acetone, an aprotic solvent, suggests that the process is primarily driven by physical mobility and the degree of macromolecular swelling rather than by active protic scavenging.

A similar increase in the *g*-values was achieved with both the swelling and thermal treatment, indicating an increase in oxygen-centered phenoxyl radicals, or a relative decrease in carbon-centered radicals, in both cases. However, solvation did not significantly alter the *T*_g_, suggesting that the two processes are not equivalent.

The mode of activation, whether solvation or thermal softening, shapes the secondary consequences (*T*_g_ change, radical-type conversion) but not the primary outcome: radical quenching. In all cases, overcoming the restricted mobility of the glassy state is the essential prerequisite for depleting persistent radicals in kraft lignin. This understanding is critical for standardizing lignin analytical protocols and for the controlled development of lignin-based thermoplastics, where radical-mediated reactions dictate the material performance. Specifically, solvent selection during lignin fractionation and drying should account for inadvertent radical quenching, which could alter reactivity in downstream thermoset cross-linking or carbon fiber stabilization processes. Equally, the *T*_g_ increase observed after thermal treatment implies that radical-mediated cross-linking is a competing reaction during melt processing that must be controlled to achieve reproducible thermoplastic properties.^3,5^

This study was limited to two solvents of contrasting protic/aprotic character; a broader solvent screen including water, THF, or DMSO would further delineate the respective contributions of protic character, polarity, and swelling power to the radical quenching efficiency. We aim to expand the scope of this article in a future publication.

## Supplementary Material



## Notes

Figure S1 showing Mn^2+^ EPR calibration standard; Figure S2 showing TM-DSC annealing thermograms, KL and MeOH-KL; Figure S3 showing ATR-FTIR spectra, KL, MeOH-KL, acetone-KL, and KL (after thermal treatment at 180 °C) (PDF)

## Abbreviations

EPR: electron paramagnetic resonance
PFRs: persistent free radicals
TM-DSC: temperature-modulated differential scanning calorimetry
DSC: differential scanning calorimetry
KL: kraft lignin
MeOH-KL: methanol-swollen kraft lignin
Acetone-KL: acetone-swollen kraft lignin
HAT: hydrogen atom transfer
CCR: carbon-centered radical
SFR: semiquinone free radical
WWSC: Wallenberg Wood Science Center
