# Supplementary material for: Understanding Lignin Radical Dynamics: Quenching Radicals by Solvent and Thermal Induced Mobility
Source: Biomacromolecules. 2026 Jun 29;27(7):4796–801. doi: 10.1021/acs.biomac.6c00744 (PMC13370768; doi:10.1021/acs.biomac.6c00744)
Supplement: Supplementary file 1 [file bm6c00744_si_001.pdf]

Supporting Information

Understanding Lignin Radical Dynamics: Quenching Radicals by Solvent and Thermal Induced Mobility.

Åke Henrik-Klemens[1,3] , Liam Mistry,[1,2] and Anette Larsson[1-3]*

[1]*Applied Chemistry, Chemistry and Chemical Engineering, Chalmers University of Technology, SE-412 96 Gothenburg, Sweden.*

[2]*Wallenberg Wood Science Center, Chalmers University of Technology, SE-412 96 Gothenburg, Sweden.*

[3]*FibRe – Centre for Lignocellulose-based Thermoplastics, Department of Chemistry and Chemical Engineering, Chalmers University of Technology, SE-412 96 Gothenburg, Sweden.*

E-Mail address: Mistry@Chalmers.se, Akehe@Chalmers.se and Anette.Larsson@Chalmers.se

Number of Pages: 3

Number of Tables: 1

Number of Figures: 3

Table of Content

**Content**	**Page No.**
EPR Standards	S2
DSC Annealing Data	S2
FTIR	S3

EPR Data

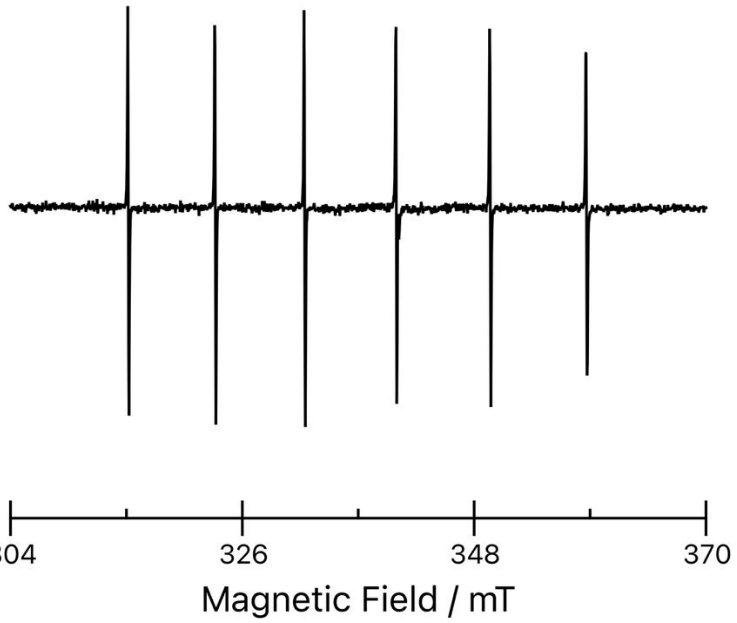

Figure S1. EPR spectra for Manganese oxide (Mn^{2+}) used as an internal reference (g-value average: 2.0021).

DSC Annealing

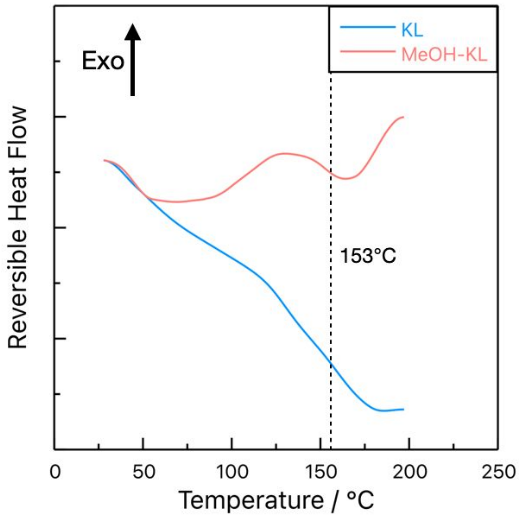

Figure S2. TM-DSC curves of treated (MeOH) and non-treated Kraft Lignin for reversible heat flow after annealing at 80°C.

Reversing heat flow during the first heating scan after annealing at 80 °C. The curve profiles are similar; however, MeOH-KL exhibits an increasing baseline slope. This difference was consistently observed across all triplicates and is likely related to differences in physical aging or structural organization due to drying process.

FTIR Spectroscopy (ATR)

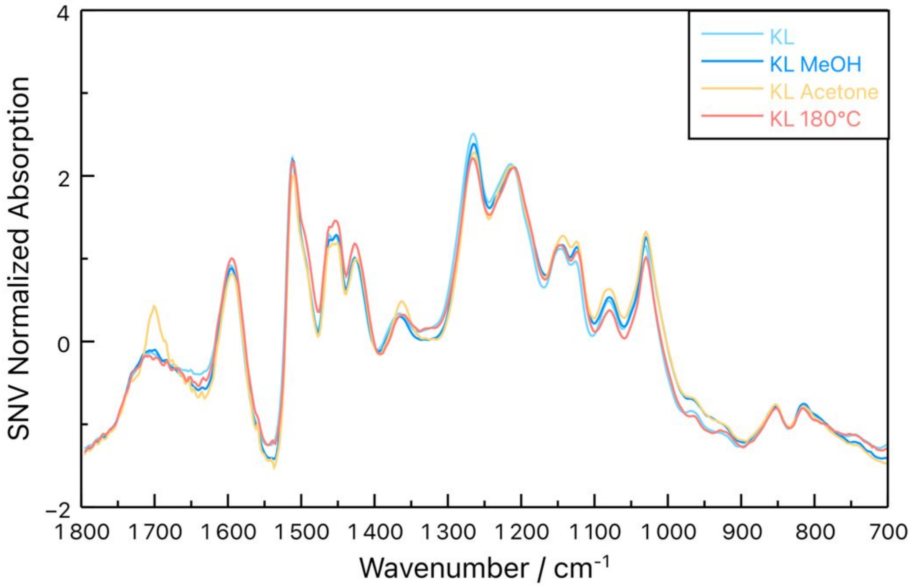

Figure S3. FTIR absorption spectra (SNV normalized) of KL, KL-MeOH, KL-Acetone and KL after thermal treatment at 180°C.

To investigate chemical changes induced by solvent and heat treatment, the samples were analyzed using a Spectrum One FTIR Spectrometer (PerkinElmer Instruments, USA) equipped with a diamond-crystal ATR accessory. Measurements were performed in five replicates, and the resulting spectra were averaged. The spectra were subsequently normalized using standard normal variate (SNV) transformation.

Termination of the condensation reactions would be expected to alter the ratio between the aromatic ring stretching at 1592 and 1510 cm^{-1}, as commonly observed for lignins with varying degrees of condensation; however, no such change was observed here.[1] Most likely, the sensitivity of FTIR is too low to detect these subtle chemical changes.

References

(1) Henrik-Klemens, Å.; Caputo, F.; Ghaffari, R.; Westman, G.; Edlund, U.; Olsson, L.; Larsson, A. The Glass Transition Temperature of Isolated Native, Residual, and Technical Lignin. *Holzforschung* **2024**, *78* (4), 216–230. https://doi.org/10.1515/hf-2023-0111.
